# Transperineal minimally invasive APE: preliminary outcomes in a multicenter cohort

**DOI:** 10.1007/s10151-020-02234-5

**Published:** 2020-06-16

**Authors:** S. E. van Oostendorp, S. X. Roodbeen, C. C. Chen, A. Caycedo-Marulanda, H. M. Joshi, P. J. Tanis, C. Cunningham, J. B. Tuynman, R. Hompes

**Affiliations:** 1 Department of Surgery, Amsterdam UMC, Vrije Universiteit, Cancer Center Amsterdam, De Boelelaan 1117, 1081 HV Amsterdam, The Netherlands; 2 Department of Surgery, Amsterdam UMC, University of Amsterdam, Cancer Center Amsterdam, Amsterdam, The Netherlands; 3 Department of Surgery, Sun Yat-Sen Cancer Center, Koo foundation, Taipei, Taiwan; 4 Department of Surgery, Health Sciences North, Sudbury, Canada; 5 Department of Colorectal Surgery, Churchill Hospital, Oxford University Hospitals NHS Foundation Trust, Oxford, UK

**Keywords:** Rectal cancer, TME, Abdominoperineal excision, Transperineal, TpAPE, ELAPE

## Abstract

**Background:**

Abdominoperineal excision (APE) for rectal cancer is associated with a relatively high risk of positive margins and postoperative morbidity, particularly related to perineal wound healing problems. It is unknown whether the use of a minimally invasive approach for the perineal part of these procedures can improve postoperative outcomes without oncological compromise. The aim of this study was to evaluate the feasibility of minimally invasive transperineal abdominoperineal excision (TpAPE)

**Methods:**

This multicenter retrospective cohort study included all patients having TpAPE for primary low rectal cancer. The primary endpoint was the intraoperative complication rate. Secondary endpoints included major morbidity (Clavien–Dindo ≥ 3), histopathology results, and perineal wound healing.

**Results:**

A total of 32 TpAPE procedures were performed in five centers. A bilateral extralevator APE (ELAPE) was performed in 17 patients (53%), a unilateral ELAPE in 7 (22%), and an APE in 8 (25%). Intraoperative complications occurred in five cases (16%) and severe postoperative morbidity in three cases (9%). There were no perioperative deaths. A positive margin (R1) was observed in four patients (13%) and specimen perforation occurred in two (6%). The unilateral extralevator TpAPE group had worse specimen quality and a higher proportion of R1 resections than the bilateral ELAPE or standard APE groups. The rate of uncomplicated perineal wound healing was 53% (*n* = 17) and three patients (9%) required surgical reintervention.

**Conclusions:**

TpAPE seems to be feasible with acceptable perioperative morbidity and a relatively low rate of perineal wound dehiscence, while histopathological outcomes remain suboptimal. Additional evaluation of the viability of this technique is needed in the form of a prospective trial with standardization of the procedure, indication, audit of outcomes and performed by surgeons with vast experience in transanal total mesorectal excision.

## Introduction

Radical surgery with adherence to the principles of total mesorectal excision (TME) is the key for local control in rectal cancer surgery [1, 2]. A meticulous TME dissection avoids involvement of the circumferential resection margin (CRM) which is directly related to local recurrence [3–5]. For low rectal cancer, achieving a complete TME is more difficult, due to tapering of the mesorectal fat surrounding the rectum in combination with limited access to the narrow bony pelvis [6–9]. To achieve safe oncological margins, tumours with threatened margins located in the low rectum are commonly subject to an abdominoperineal excision (APE) [10, 11].

Despite more extensive surgery in which the anus and sphincter complex are excised en-bloc with the rectum, the clinical and oncological outcomes after APE are far from optimal. As shown in the Dutch TME trial, the rate of involved CRM (CRM +) was substantially higher for APE compared to anterior resection, 30.4% vs 10.7% (*p* = 0.002) respectively [12]. Coning of the specimen towards the pelvic floor with a “waist” at the puborectal sling was put forward as the main culprit for the higher CRM + rates and formed the rationale for a cylindrical excision [13]. In this so called extralevator APE (ELAPE), a wider distal dissection route is followed which includes en-bloc excision of the levator ani muscles leading to a lower rate of CRM + and tumour perforation [13–16]. The wider and thereby more radical excision comes at the cost of a larger defect of the pelvic floor and skin [17–20]. Previous studies and meta-analyses have reported major morbidity rates between 10–30% and perineal wound healing problems from 11 up to 50% [21–26]. Especially in irradiated patients, perineal wound healing is problematic and sometimes requires primary or secondary reconstruction with musculocutaneous flaps to achieve perineal closure [26–30].

A minimally invasive transperineal approach to the perineal part of an APE has potential advantages over the standard technique, although data on this new technique are limited [33]. This multicenter series describes the combined initial experience of five expert centers in four countries with a transperineal minimally invasive APE technique (TpAPE) for locally advanced low rectal cancer. The primary aim was to assess the feasibility by reporting on intraoperative complications. Secondary aims were to assess the histopathological outcomes and postoperative morbidity including the incidence and management of perineal wound complications.

## Materials and methods

### Patients

A consecutive cohort of patients who underwent TpAPE for primary rectal cancer was identified at five centers (two in The Netherlands, one in Taiwan, one in Canada, and one in the United Kingdom). This group consisted of patients that had either a bilateral ELAPE, an unilateral ELAPE or APE with resection of the entire external sphincter [31, 32]. Intersphincteric APE’s were excluded. A retrospective analysis of prospective institutional databases was performed, and individual patient data were provided by an anonymized data sheet. The annual volume of rectal cancer surgery varied amongst the participating centers, but all perform over 50 resections (including transanal minimally invasive local excision, partial mesorectal excision, low anterior resection, APE, ELAPE, and recurrent rectal cancer).

### Surgical technique

The patient is placed in a lithotomy position to enable simultaneous abdominal and perineal dissection. The abdominal phase is performed by a standard laparoscopic medial to lateral mobilization of the left colon. The inferior mesenteric vein is ligated near the lower border of the pancreas and the inferior mesenteric artery ligated with preservation of the left colic artery. The mesorectal plane is opened with autonomous nerve preservation and dissection is continued up to connection with the perineal team. The perineal phase commences with a purse string closure of the anus. Afterwards, a radial perineal incision at approximately 1 cm from the closed anus is made into the subcutaneous fat. A single port can be inserted after creating a 2–3 cm deep opening of the ischioanal fat around external sphincter and connected to a continuous high flow insufflation and smoke exfiltration system. The most frequently used single port devices are the Gelpoint Mini and Gelpointh path (Applied Medical, Rancho Santa Margarita, California, USA). Standard laparoscopic instruments including a diathermic hook or spatula are needed for the endoscopic perineal phase. Dissection continues cephalad until the pelvic floor (levator ani muscle) is reached. Continuation externally along the levator ani muscle is tailored on a case by case level. For uni- or bilateral ELAPE procedures, the pelvic floor is followed on one or both sides up to the fascia of the obturator internus muscle and transected at this level. For a standard APE, the pelvic floor is usually transected a few centimeters out from the puborectal sling. The transection usually starts at the level of the coccyx and thereafter going forward. By cutting the pelvic floor muscle, and the overlying pelvic floor fascia connection is made with the abdominal team without coning in on the tumor. Identification of the correct anterior plane, remains the most difficult step. It is crucial to identify the transverse perineal muscles to enter this plane just posteriorly to these muscle fibres to find the avascular plane in front of the posterior vaginal wall or prostate and then continuous cephalad in front or behind Denonvillier’s fascia pending anterior location of the tumour. The specimen is extracted trough the perineal wound. The perineal defect is then closed primarily, with a subcutaneous gluteal turnover flap [33] or by aid of (biological) mesh upon individual basis.

### Outcomes

The primary endpoint was feasibility of the technique in terms of intraoperative complications.[34]. Secondary endpoints included 30-day major morbidity (Clavien Dindo ≥ 3), perineal wound healing, and histopathological outcomes. CRM + was defined as presence of tumour cells ≤ 1 mm of the surgical plane. The specimen quality was graded according to Quirke [2]. Perforation was defined as a tear or hole from the surface of the surgical specimen (mesorectum or at the level of the sphincters) into the rectal lumen.

### Statistical analysis

All data are presented as *N* (%) for binary data and for continuous outcome as mean ± SD and median range as well since normal distribution is not expected in this small cohort. To explore the potential impact for the extent of the procedure, which increases from APE via an unilateral ELAPE to a bilateral ELAPE, a comparative analysis for these procedures was performed. For the comparative analysis, a Fisher’s exact test was used for categorical variables and the non-parametric Mann–Whitney *U* test or Kruskal–Wallis test for continuous variables. A *p* value < 0.05 was considered statistically significant. Statistical analysis was performed using SPSS version 24 for Windows (SPSS, Chicago, IL, USA).

## Results

### Baseline characteristics (Table 1)

**Table 1:** Patient and tumour characteristics

		Transperineal APE *N* = 32
Sex	Male (%)	24 (75.0)
	Female (%)	8 (25.0)
BMI kg/m^2^ (mean) (± SD)		26.4 (3.3)
Age,years (mean) (± SD)		65.7 (12.8)
ASA class	I	1 (3.1)
	II	25 (78.1)
	III	6 (18.8)
Tumour height from ARJ (cm)*	Mean (± SD)	0.50 (0.87)
	Median (range)	0 (0–3.0)
T-stage (%)	cTis	1 (3.1)
	cT2	7 (21.9)
	cT3	17 (53.1)
	cT4b	7 (21.9)
N-stage (%)	N0	18 (56.3)
	N1	8 (25.0)
	N2	6 (18.8)
M-stage (%)	M +	1 (3.1)**
Mesorectal fascia involved	Yes	21 (65.6)
	No	11 (34.4)
EMVI	Yes	8 (25.0)
	No	18 (56.3)
	Unknown	6 (18.8)
Ingrowth	No	16 (50.0)
	Spinctercomplex	10 (31.3)
	M. levator ani	4 (12.5)
	Prostate / vagina	2 (6.3)
Neoadjuvant radiotherapy (%)	No	9 (28.1)
	5 × 5 short interval	3 (9.4)
	long course Chemorad	20 (62.5)

Numbers in parenthesis are percentages, unless mentioned otherwise

*BMI* body mass index, *SD* standard deviation, *ASA* American Society of Anesthesiologists, *RT* radiotherapy, *CRT* chemoradiotherapy, *ARJ* anorectal junction

^*^22 of 32 located at or below level of ARJ

^**^Para-aortic M +

A total of 32 patients were included (24 males, mean age 65.7 [± 12.8]) from 5 different expert colorectal cancer centers with a case load varying between 1 and 12 procedures. The first procedure in this series was performed in June 2014 and the last in July 2018. Seventeen patients had cT3 stage rectal cancer and seven were cT4 stage (Table 1). Nodal involvement was diagnosed in 14 patients (44%). In one patient, a suspected para-aortic lymph node metastasis was present and, therefore, categorized as distant (M +) disease. No other peritoneal, liver or lung metastasis were encountered in the preoperative work-up. The majority of cases was found to have a threatened margin to the mesorectal fascia (MRF) (*n* = 12, 66%) on baseline magnetic resonance imaging (MRI) and the tumour was located at or below the level of the anorectal junction in 22 cases (69%). Extension of low tumours into the sphincter complex was seen in ten patients, ingrowth into the levator ani muscles and anterior involvement (prostate or vagina) was encountered in four and two cases, respectively. A substantial part of the patients received neoadjuvant chemoradiotherapy (*n* = 20, 63%).

A bilateral extralevatory APE was performed in 17 cases (53%), a unilateral ELAPE in 7 cases (22%), and an APE without resection of the levator ani muscle in 8 patients(25%) (Table 2). A beyond TME resection (*n* = 8, 25%) was performed for tumours that invaded other organs or those at risk of CRM +, and consisted of additional (partial) resection of the prostate (*n* = 5), vagina (*n* = 1), seminal vesicles (*n* = 1), and ovaries (*n* = 1). An omentoplasty was performed in six patients (19%), all without use of indocyanine green, to assess the perfusion of the mobilized greater omentum. The perineal defect was predominantly closed by primary closure (*n* = 18, 56%), and in the other 15 cases, either a musculocutaneous gluteal flap (*n* = 6, 19%), an absorbable mesh (*n* = 7, 22%) or a non-absorbable mesh (*n* = 1, 3%) was used for perineal reconstruction.

**Table 2:** Operative details

		Transperineal APE *N* = 32
Type of surgery	APE, levators left in situ	8 (25.0)
	Unilateral ELAPE	7 (21.9)
	Bilateral ELAPE	17 (53.1)
Beyond TME resection	No	24 (75.0)
	Prostate/Vagina	6 (18.8)
	Seminal vesicles	1 (3.1)
	Ovaries	1 (3.1)
	Pelvic sidewall	0 (0)
Operative time (min)	Mean (± SD)	278 (78)
	Median (range)	249 (175–450)
Blood loss (ml)	Mean (± SD)	203 (115)
	Median (range)	200 (50–400)
Conversion	To open perineal dissection	1 (3.1)
	To laparotomy	0 (0)
Intraoperative events	Urethral injury	1 (3.1)
	Pelvic sidewall injury	1 (3.1)
	Co_2_ embolism	1 (3.1)
	Rectal tube perforation	2 (6.3)
Omentoplasty performed	Yes	6 (18.8)
Perineal reconstruction	Primary closure	18 (56.3)
	Gluteal turnover flap*	3 (9.4)
	Gluteus maximus flap**	3 (9.4)
	Non-absorbable mesh	1 (3.1)
	Absorbable mesh	7 (21.9)

Numbers in parenthesis are percentages, unless mentioned otherwise

*APE* Abdominoperineal excision, *ELAPE* extralevator abdominoperineal excision, *TME* total mesorectal excision

^*^Deepithelialized cutaneous turnover flap, **musculocutaneous flap

### Primary endpoint

Intraoperative complications occurred in five patients (16%) and consisted of one carbon dioxide embolus, one urethral injury, one pelvic sidewall injury and two intraoperative rectal perforations. No conversions to laparotomy were reported and conversion to an open perineal approach was necessary once, due to the inability to progress with the dissection, despite abdominal assistance in a two-team approach (Table 2).

### Secondary endpoints

There was no 30-day mortality. Major 30-day postoperative morbidity was reported in three patients (9%). This consisted of a compartment syndrome of the lower leg requiring fasciotomy, a deep pelvic abscess due to omental infarction with return to theatre and a urinoma following urethral injury which was managed with percutaneous drainage (Table 3). Perineal wound healing was impaired in 47% (*n* = 15) of patients in this cohort; one flap failure (3%), four break through abscesses (deep perineal infection) (13%), and ten superficial skin infections (31%). One superficial dehiscence and one flap failure were treated by negative pressure therapy, three breakthrough abscesses needed packing, and one abscess required drainage followed by secondary healing. One patient with a superficial skin infection that was initially not severe developed a late perineal hernia with wound dehiscence requiring secondary reconstruction. The median time to perineal wound healing was 14 days for those without perineal infection and 45 days in complicated perineal recovery (*p* = 0.002).

**Table 3:** Postoperative details

		Transperineal APE *N* = 32
Mortality (30 day)		0 (0)
Total postoperative complications CD	None	11 (34.4)
	Minor (CD I-II)	18 (56.3)
	Major (CD ≥ III)	3 (9.4)
Perineal wound healing	uncomplicated	17 (53.1)
	complicated	15 (46.9)
Nature of healing complications	Superficial infection	10 (31.3)
	Break through abscess	4 (12.5)
	Flap failure	1 (3.1)
Days to heal (days) median (range)	Uncomplicated	14 (5–60)
	Complicated	45 (21–140)

Numbers in parenthesis are percentages, unless mentioned otherwise

*APE* Abdominoperineal excision

CRM + (R1) upon pathological evaluation was seen in four cases (13%) and intraoperative specimen perforation occurred in two procedures (6%). The positive margin was anterior in three out of the four R1 resections. A complete or nearly complete specimen was obtained in the vast majority of cases (*n* = 28, 90%) (Table 4).

**Table 4:** Pathological assessment

		Transperineal APE *N* = 32
Pathology stage	(y)pT0	4 (12.5)
	(y)pT1	0 (0)
	(y)pT2	9 (28.1)
	(y)pT3	19 (59.4)
Successful resection	Yes	5 (15.6)
	No	27 (84.4)
Quality of specimen (Quirke)*	Complete	15 (48.4)
	Nearly complete	13 (41.9)
	Incomplete	3 (9.7)
CRM involvement (< 1 mm)	Yes	4 (12.5)
	No	28 (87.5)
Perforation	Yes	2 (6.3)
	No	30 (93.8)
Lymph nodes harvested	Mean (± SD)	13.6 (7.9)
	Median (range)	12 (2–34)
Pathologic N stage	(y)pN0	20 (62.5)
	(y)pN1	9 (28.1)
	(y)pN2	3 (9.4)

Numbers in parenthesis are percentages, unless mentioned otherwise

*APE* Abdominoperineal excision, *CRM* circumferential resection margin

*1 missing

### Comparative analysis

Comparative analysis based on the extent of the procedure (conventional APE, uni- or bilateral ELAPE) revealed that intraoperative complications were higher in the ELAPE groups (Table 5). There were three severe complications in the unilateral ELAPE (pelvic sidewall injury, urethral injury, and rectal tube perforation), two in the bilateral ELAPE (CO_2_ embolus and rectal tube perforation), and no intraoperative complications in the APE group (*p* 0.071). Severe postoperative complications were distributed equally among the three procedures. A composite of optimal pathology, defined as circumferential and distal resection margin-without perforation and a complete/near complete specimen, was achieved in 84.4% cases in this series. An unsuccessful resection was seen in two out of seven (29%) unilateral ELAPE procedures which was higher than in conventional APE (13%) and bilateral ELAPE (16%) but did not reach statistical significance.

**Table 5:** Comparative

		APE *n* = 8	Unilateral ELAPE *n* = 7	Bilateral ELAPE *n* = 17	*p* value
Baseline					
Sex	Male (%)	7 (87.5)	6 (85.7)	11 (64.7)	0.488
	Female (%)	1 (12.5)	1 (14.3)	6 (35.3)	
Age, years	Mean (± SD)	70.3 (7.1)	69.3 (13.7)	62.0 (14.0)	0.241
	Median (range)	71 (55–80)	70.0 (47–86)	63.0 (33–83)	
BMI kg/m^2^	Mean (± SD)	28.3 (2.5)	25.0 (3.6)	26.1 (3.3)	0.121
	Median (range)	28.2 (25.5–31.5)	24.9 (20.3–30.7)	25.6 (20.2–33.0)	
ASA class	< III	7 (87.5)	4 (57.3)	15 (88.2)	0.202
	≥ III	1 (12.5)	3 (42.7)	2 (11.8)	
Tumour stage (cT)	≤ T3	7 (87.5)	3 (42.7)	15 (88.2)	0.054
	T4	1 (12.5)	4 (57.3)	2 (11.8)	
Mesorectal fascia threatened	Yes (%)	3 (37.5)	6 (85.7)	12 (70.6)	0.146
	No (%)	5 (62.5)	1 (14.3)	5 (29.4)	
Height with respect to ARJ	At or below (%)	6 (75.0)	2 (28.6)	14 (82.4)	**0.045**
	Above (%)	2 (25.0)	5 (71.4)	3 (17.6)	
Intraoperative outcomes					
Operative time (minutes)	Mean (± SD)	256 (50)	245 (50)	297 (83)	0.250
	Median (range)	242 (175–300)	245 (175–300)	300 (180–450)	
Intraoperative complications	Yes (%)	0 (0)	3 (42.9)	2 (11.8)	0.071
	No (%)	8 (100)	4 (57.3)	14 (82.4)	
Pathological outcomes					
Quality of specimen (Quirke)	Complete	6 (75.0)	5 (71.4)	4 (25.0)	**0.022**
	Nearly complete	1 (12.5)	1 (14.3)	11 (68.8)	
	Incomplete	1 (12.5)	1 (14.3)	1 (6.3)	
CRM involvement	Yes (%)	1 (12.5)	2 (28.6)	1 (5.9)	0.306
	No (%)	7 (87.5)	5 (71.4)	16 (94.1)	
Perforation	Yes (%)	0 (0)	2 (28.6)	0 (0)	**0.042**
	No (%)	8 (100)	5 (71.4)	17 (100)	
Successful resection	Yes (%)	7 (87.5)	5 (71.4)	15 (84.4)	0.679
	No (%)	1 (12.5)	2 (28.6)	2 (15.6)	
Postoperative outcomes					
Severe 30 day morbidity (CD ≥ 3)	Yes (%)	1 (12.5)	1 (14.3)	1 (5.9)	0.781
	No (%)	7 (87.5)	6 (85.7)	16 (94.1)	
Days to perineal wound healing	Mean (± SD)	44.0 (39.0)	25.8 (16.6)	51.2 (36.9)	0.354
	Median (range)	33 (5–106)	22 (7–45)	42 (6–140)	
Perineal wound healing	Uncomplicated	3 (37.5)	4 (57.1)	10 (58.8)	0.709
	Superficial infection	5 (50.0)	1 (14.3)	5 (29.4)	
	Omental/Break- through abscess	1 (12.5)	2 (28.6)	1 (5.9)	
	Flap failure	0 (0)	0 (0)	1 (5.9)	

Numbers in parenthesis are percentages, unless mentioned otherwise

Significant *p* values are in bold (*p* < 0.05)

*CD* Clavien–Dindo, *n.p.* not performed, *CRM* circumferential resection margin, *APE* abdominoperineal excidsion, *ELAPE* extralevator abdoiminoperineal excision, *ARJ* anorectal junction, *ASA* American Society of Anesthesiologists, *BMI* body mass index

## Discussion

This multicenter case series suggests that minimally invasive TpAPE is feasible with acceptable intraoperative complications, no short-term mortality and a 9% severe postoperative complication rate within 30 days.

The postoperative major morbidity rate of 9% compares favorably to major morbidity rates between 10 and 30% and perineal wound infection ranging from 11 up to 50% reported in large series and meta-analysis, but the current study is limited by the small sample size and inherent case selection bias [21–26]. Five intraoperative complications were reported, four of which were related to wrong plane surgery with sequential perforation, urethral, and pelvic sidewall injury. This illustrates the complexity of this technique and further evaluation of safety and development of the technique are warranted.

The minimally invasive transperineal approach with the application of a single port diminishes the need for a large perineal skin incision to facilitate sufficient exposure to complete the extra-sphincteric dissection and resection of the pelvic floor as required. The down-to-up approach offers good visualization and access to the extralevator plane and does not require rotation of the patient to a prone position and/or resection of the coccyx to complete the posterior plane. In addition, the anterior plane between the specimen and the prostate or vagina can be dissected endoscopically which prevents externalization and rotation of the specimen.

Using the conventional open approach for an APE, perineal wound breakdown is a major issue as summarized in a meta-analysis of Musters et al*.* [35]. Impaired perineal healing after primary closure occurred in 15.3% of APE and 14.8% of ELAPE procedures, both without neoadjuvant treatment, which increased to 30.2% and 37.6%, respectively, for APE and ELAPE with neoadjuvant radiotherapy [35]. Moreover, in the LOREC APE registry (UK), up to 31% perineal wound breakdown for APE and ELAPE was encountered [23]. Dehiscence often requires intensive treatment with prolonged wound packing, vacuum therapy and in case of pelvic sepsis, image-guided percutaneous drainage [36], which is reflected in a substantial increase in length of stay, readmission rate, and costs [26, 37]. In the current series, in 5 patients (16%), a breakthrough abscess or flap failure occurred, and 3 out of 32 patients (9%) needed a surgical reintervention under general anesthesia for a perineal wound complication. Interestingly, in addition to the aforementioned patients, 12 other patients developed a superficial skin infection which could be managed conservatively, i.e. by dressings, antibiotics, or removal of sutures. Due to the reduced length of the incision, a superficial infection after a minimally invasive transperineal approach is probably less likely to culminate in a complete breakdown of the perineal area which seems to occur more frequently after a conventional open perineal approach.

The introduction of ELAPE by Holm et al*.* in [13] has shown potential to decrease the rate of intraoperative tumour perforation and CRM + rates [13, 14, 19]. Randomized data only comes from small randomized clinical trials and supports the potential oncologic benefit of reduced R1 resection in ELAPE [18, 38]. Future larger size trials are awaited to add more robust data, especially on tailoring the extent of surgery to uni- or bilateral ELAPE. In this series, CRM + was more frequently encountered in unilateral ELAPE than APE or bilateral ELAPE: 29% versus 13% and 6%, respectively, (*p* = 0.306). Intraoperative tumour perforation occurred twice, both in unilateral ELAPE. In three out of four R1 resections, the positive margin was found in the anterior dissection plane which shows that also in an extensive proctectomy, an anterior tumour location is at high risk of a positive margin. This is in line with data from the Mercury II study and transanal total mesorectal excision (TaTME) registry and, therefore, these cases should not be performed early in the learning curve [9, 39]. In retrospect, an anterior exenteration with en-bloc resection of the posterior vaginal wall or prostate might have been more suitable. Eliminating these cases provides an acceptable involved margin rate of 3% (1 out of 29).

Comparable results regarding this technique were reported by Yasukawa et al., who described a comparative cohort of 21 minimally invasive TpAPE versus 29 conventional APE with a positive margin rate (2/21 versus 3/29), a lower severe perineal wound infection rate (0/21 versus 5/29) and reduced length of stay (median 14 versus 23 days) with no conversion, no mortality, and no increase in major morbidity [31].

The current study is limited by several factors that result from its design. With a total of 32 cases from 5 large rectal cancer referral centers, selection bias is indisputably present. Furthermore, the learning curve is likely to partly explain the suboptimal outcomes [40]. Although all the surgeons were highly experienced in TaTME, the extension of the down-to-up approach to Tp (EL) APE adds to the procedural complexity. In addition, with institutional variation in treatment algorithms for both initial resection and management of complications including variety in follow-up protocols, further standardized studies are warranted with appropriate institutional review board approval. In particular, since this technique is promising regarding wound healing and recovery, standardized registration of time to perineal wound healing is essential. However, before initiation of (larger) studies on the potential improvement in perineal wound healing, further evaluation should focus on the safety in terms of intraoperative morbidity and oncologic safety within a prospective well-designed trial.

## Conclusions

Tp(EL)APE seems to be feasible with acceptable perioperative morbidity and a low rate of perineal wound dehiscence, while histopathological outcomes remain suboptimal. High complexity necessitates extensive experience in both TaTME and conventional ELAPE. Additional evaluation of this technique is needed, ideally in the form of a prospective trial with standardization of the procedure, indications and prospective audited data collection to further explore the safety and viability of this technique.

## References

[1] Heald RJ, Ryall RD (1986). Recurrence and survival after total mesorectal excision for rectal cancer. Lancet.

[2] Quirke P, Steele R, Monson J, Grieve R, Khanna S, Couture J, O'Callaghan C, Myint AS, Bessell E, Thompson LC, Parmar M, Stephens RJ, Sebag-Montefiore D, Investigators MCN-CCT, Group NCCS (2009). Effect of the plane of surgery achieved on local recurrence in patients with operable rectal cancer: a prospective study using data from the MRC CR07 and NCIC-CTG CO16 randomised clinical trial. Lancet.

[3] Nagtegaal ID, Quirke P (2008). What is the role for the circumferential margin in the modern treatment of rectal cancer?. J Clin Oncol.

[4] Bonjer HJ, Deijen CL, Abis GA, Cuesta MA, van der Pas MH, de Lange-de Klerk ES, Lacy AM, Bemelman WA, Andersson J, Angenete E, Rosenberg J, Fuerst A, Haglind E, Group CIS (2015). A randomized trial of laparoscopic versus open surgery for rectal cancer. N Engl J Med.

[5] Taylor FG, Quirke P, Heald RJ, Moran B, Blomqvist L, Swift I, St Rose S, Sebag-Montefiore DJ, Tekkis P, Brown G, Group Ms (2011). One millimetre is the safe cut-off for magnetic resonance imaging prediction of surgical margin status in rectal cancer. Br J Surg.

[6] Taylor FG, Quirke P, Heald RJ, Moran B, Blomqvist L, Swift I, Sebag-Montefiore DJ, Tekkis P, Brown G, Group Ms (2011). Preoperative high-resolution magnetic resonance imaging can identify good prognosis stage I, II, and III rectal cancer best managed by surgery alone: a prospective, multicenter European study. Ann Surg.

[7] Salerno G, Sinnatamby C, Branagan G, Daniels IR, Heald RJ, Moran BJ (2006). Defining the rectum: surgically, radiologically and anatomically. Colorectal Dis.

[8] How P, Shihab O, Tekkis P, Brown G, Quirke P, Heald R, Moran B (2011). A systematic review of cancer related patient outcomes after anterior resection and abdominoperineal excision for rectal cancer in the total mesorectal excision era. Surg Oncol.

[9] Battersby NJ, How P, Moran B, Stelzner S, West NP, Branagan G, Strassburg J, Quirke P, Tekkis P, Pedersen BG, Gudgeon M, Heald B, Brown G, Group MIS (2016). Prospective validation of a low rectal cancer magnetic resonance imaging staging system and development of a local recurrence risk stratification model: the MERCURY II study. Ann Surg.

[10] Shihab OC, Brown G, Daniels IR, Heald RJ, Quirke P, Moran BJ (2010). Patients with low rectal cancer treated by abdominoperineal excision have worse tumors and higher involved margin rates compared with patients treated by anterior resection. Dis Colon Rectum.

[11] Miles WE (1971). A method of performing abdomino-perineal excision for carcinoma of the rectum and of the terminal portion of the pelvic colon (1908). CA Cancer J Clin.

[12] Nagtegaal ID, van de Velde CJ, Marijnen CA, van Krieken JH, Quirke P, Dutch Colorectal Cancer G, Pathology Review C (2005). Low rectal cancer: a call for a change of approach in abdominoperineal resection. J Clin Oncol.

[13] Holm T, Ljung A, Haggmark T, Jurell G, Lagergren J (2007). Extended abdominoperineal resection with gluteus maximus flap reconstruction of the pelvic floor for rectal cancer. Br J Surg.

[14] Stelzner S, Koehler C, Stelzer J, Sims A, Witzigmann H (2011). Extended abdominoperineal excision vs standard abdominoperineal excision in rectal cancer—a systematic overview. Int J Colorectal Dis.

[15] Dutch Snapshot Research G (2017). Benchmarking recent national practice in rectal cancer treatment with landmark randomized controlled trials. Colorectal Dis.

[16] van Leersum N, Martijnse I, den Dulk M, Kolfschoten N, Le Cessie S, van de Velde C, Tollenaar R, Wouters M, Rutten HJ (2014). Differences in circumferential resection margin involvement after abdominoperineal excision and low anterior resection no longer significant. Ann Surg.

[17] Holm T (2017). Abdominoperineal excision: technical challenges in optimal surgical and oncological outcomes after abdominoperineal excision for rectal cancer. Clin Colon Rectal Surg.

[18] Han JG, Wang ZJ, Wei GH, Gao ZG, Yang Y, Zhao BC (2012). Randomized clinical trial of conventional versus cylindrical abdominoperineal resection for locally advanced lower rectal cancer. Am J Surg.

[19] West NP, Finan PJ, Anderin C, Lindholm J, Holm T, Quirke P (2008). Evidence of the oncologic superiority of cylindrical abdominoperineal excision for low rectal cancer. J Clin Oncol.

[20] Huang A, Zhao H, Ling T, Quan Y, Zheng M, Feng B (2014). Oncological superiority of extralevator abdominoperineal resection over conventional abdominoperineal resection: a meta-analysis. Int J Colorectal Dis.

[21] Aggarwal N, Seshadri RA, Arvind A, Jayanand SB (2018). Perineal wound complications following extralevator abdominoperineal excision: experience of a regional cancer center. Indian J Surg Oncol.

[22] Blok RD, de Jonge J, de Koning MA, van de Ven AWH, van der Bilt JDW, van Geloven AAW, Hompes R, Bemelman WA, Tanis PJ (2019). Propensity score adjusted comparison of pelviperineal morbidity with and without omentoplasty following abdominoperineal resection for primary rectal cancer. Dis Colon Rectum.

[23] Jones H, Moran B, Crane S, Hompes R, Cunningham C, Group L (2017). The LOREC APE registry: operative technique, oncological outcome and perineal wound healing after abdominoperineal excision. Colorectal Dis.

[24] Habr-Gama A, Sao Juliao GP, Mattacheo A, de Campos-Lobato LF, Aleman E, Vailati BB, Gama-Rodrigues J, Perez RO (2017). Extralevator abdominal perineal excision versus standard abdominal perineal excision: impact on quality of the resected specimen and postoperative morbidity. World J Surg.

[25] Yang XY, Wei MT, Yang XT, He YZ, Hao Y, Zhang XB, Deng XB, Wang ZQ, Zhou ZQ (2019). Primary vs myocutaneous flap closure of perineal defects following abdominoperineal resection for colorectal disease: a systematic review and meta-analysis. Colorectal Dis.

[26] Musters GD, Sloothaak DA, Roodbeen S, van Geloven AA, Bemelman WA, Tanis PJ (2014). Perineal wound healing after abdominoperineal resection for rectal cancer: a two-centre experience in the era of intensified oncological treatment. Int J Colorectal Dis.

[27] Prytz M, Angenete E, Ekelund J, Haglind E (2014). Extralevator abdominoperineal excision (ELAPE) for rectal cancer–short-term results from the Swedish Colorectal Cancer Registry. Selective use of ELAPE warranted. Int J Colorectal Dis.

[28] Peirce C, Martin S (2016). Management of the perineal defect after abdominoperineal excision. Clin Colon Rectal Surg.

[29] Bullard KM, Trudel JL, Baxter NN, Rothenberger DA (2005). Primary perineal wound closure after preoperative radiotherapy and abdominoperineal resection has a high incidence of wound failure. Dis Colon Rectum.

[30] Foster JD, Tou S, Curtis NJ, Smart NJ, Acheson A, Maxwell-Armstrong C, Watts A, Singh B, Francis NK (2018). Closure of the perineal defect after abdominoperineal excision for rectal adenocarcinoma—ACPGBI Position Statement. Colorectal Dis.

[31] Yasukawa D, Hori T, Kadokawa Y, Kato S, Aisu Y, Hasegawa S (2018). Trans-perineal minimally invasive surgery during laparoscopic abdominoperineal resection for low rectal cancer. Surg Endosc.

[32] Buchs NC, Kraus R, Mortensen NJ, Cunningham C, George B, Jones O, Guy R, Ashraf S, Lindsey I, Hompes R (2015). Endoscopically assisted extralevator abdominoperineal excision. Colorectal Dis.

[33] Blok RD, Hagemans JAW, Burger JWA, Rothbarth J, van der Bilt JDW, Lapid O, Hompes R, Tanis PJ (2019). Feasibility of a subcutaneous gluteal turnover flap without donor site scar for perineal closure after abdominoperineal resection for rectal cancer. Tech Coloproctol.

[34] Dindo D, Demartines N, Clavien PA (2004). Classification of surgical complications: a new proposal with evaluation in a cohort of 6336 patients and results of a survey. Ann Surg.

[35] Musters GD, Buskens CJ, Bemelman WA, Tanis PJ (2014). Perineal wound healing after abdominoperineal resection for rectal cancer: a systematic review and meta-analysis. Dis Colon Rectum.

[36] Blackham AU, Sanchez J, Shibata D, Chang GJ (2018). Abdominoperineal excision. Rectal cancer: modern approaches to treatment.

[37] Wiatrek RL, Thomas JS, Papaconstantinou HT (2008). Perineal wound complications after abdominoperineal resection. Clin Colon Rectal Surg.

[38] Bianco F, Romano G, Tsarkov P, Stanojevic G, Shroyer K, Giuratrabocchetta S, Bergamaschi R, International Rectal Cancer Study G (2017). Extralevator with vs nonextralevator abdominoperineal excision for rectal cancer: the RELAPe randomized controlled trial. Colorectal Dis.

[39] Roodbeen SX, de Lacy FB, van Dieren S, Penna M, Ris F, Moran B, Tekkis P, Bemelman WA, Hompes R, International Ta TMERC (2019). Predictive factors and risk model for positive circumferential resection margin rate after transanal total mesorectal excision in 2653 patients with rectal cancer. Ann Surg.

[40] Koedam TWA, Veltcamp Helbach M, van de Ven PM, Kruyt PM, van Heek NT, Bonjer HJ, Tuynman JB, Sietses C (2018). Transanal total mesorectal excision for rectal cancer: evaluation of the learning curve. Tech Coloproctol.

